# A Sensitive and Accurate Recombinase Polymerase Amplification Assay for Detection of the Primary Bacterial Pathogens Causing Bovine Respiratory Disease

**DOI:** 10.3389/fvets.2020.00208

**Published:** 2020-04-22

**Authors:** Cheyenne C. Conrad, Rana K. Daher, Kim Stanford, Kingsley K. Amoako, Maurice Boissinot, Michel G. Bergeron, Trevor Alexander, Shaun Cook, Brenda Ralston, Rahat Zaheer, Yan D. Niu, Tim McAllister

**Affiliations:** ^1^Agriculture and Agri-Food Canada, Lethbridge Research and Development Centre, Lethbridge, AB, Canada; ^2^Centre de Recherche en Infectiologie de l'Université Laval, Québec City, QC, Canada; ^3^Alberta Agriculture and Forestry, Lethbridge, AB, Canada; ^4^National Centre for Animal Disease, Canadian Food Inspection Agency, Lethbridge, AB, Canada; ^5^Alberta Agriculture and Forestry, Airdrie, AB, Canada; ^6^Faculty of Veterinary Medicine, University of Calgary, Calgary, AB, Canada

**Keywords:** recombinase polymerase amplification, bovine respiratory disease, antimicrobial resistance, integrative conjugative element, competitive internal amplification

## Abstract

Rapid and accurate diagnosis of bovine respiratory disease (BRD) presents a substantial challenge to the North American cattle industry. Here we utilize recombinase polymerase amplification (RPA), a fast and sensitive isothermal DNA-based technology for the detection of four BRD pathogens (*Mannheimia haemolytica, Pasteurella multocida, Histophilus somni, Mycoplasma bovis*), genes coding antimicrobial resistance (AMR) and integrative conjugative elements (ICE) which can harbor AMR genes. Eleven RPA assays were designed and validated including: a) one conventional species-specific multiplex assay targeting the 4 BRD pathogens, b) two species-specific real-time multiplex RPA assays targeting *M. haemolytica*/*M. bovis* and *P. multocida*/*H. somni*, respectively with a novel competitive internal amplification control, c) seven conventional assays targeting AMR genes (*tetH, tetR, msrE, mphE, sul2, floR, erm42*), and d) one real-time assay targeting ICE. Each real-time RPA assay was tested on 100 deep nasopharyngeal swabs (DNPS) collected from feedlot cattle previously assessed for targets using either culture methods and/or polymerase chain reaction (PCR) verification (TC-PCR). The developed RPA assays enabled sensitive and accurate identification of BRD agents and AMR/ICE genes directly from DNPS, in a shorter period than TC-PCR, showing considerable promise as a tool for point-of-care identification of BRD pathogens and antimicrobial resistance genes.

## Introduction

Bovine respiratory disease (BRD) remains the most common and economically important disease affecting feedlot cattle, veal calves, weaned dairy heifers and beef calves (1, 2). Approximately 15% of cattle in North America are treated for BRD, accounting for 70% of morbidities and 40% of all cattle mortalities in feedlots (3, 4). Economic losses to the United States feedlot industry have been reported to be as high as 1 billion dollars annually, due to losses in production, increased labor expenses, drug costs, and death (5, 6). As the clinical symptoms associated with BRD may be non-specific, subtle and exhibit a rapid onset, fast and accurate diagnosis of BRD presents a significant challenge (2). Often, cattle with BRD are detected late in the disease process or not at all (2).

BRD is characterized by complex interactions between the host's immune system, bacterial (i.e., *Mannheimia haemolytica, Pasteurella multocida, Histophilus somni*, and *Mycoplasma bovis*) and viral (i.e., Bovine Herpes Virus-1, Parainfluenza-3, Bovine Viral Diarrhea Virus, Bovine Respiratory Syncytial Virus) pathogens and management practices that increase stress such as weaning and transportation (4, 6–8). Although *M. haemolytica* is considered to be the predominant BRD agent (9), many of the bacterial species involved are ubiquitous and considered to be commensals of the bovine respiratory tract of healthy animals (4). However, suppression of the host immune system as a result of stress or viral infection can allow these pathogens to proliferate within the upper respiratory tract, spreading to the lower respiratory tract, resulting in lesions and acute pleuropneumonia (4, 6).

Controlling BRD is the primary reason for the use of antimicrobials in feedlot cattle (4). Often, metaphylactic administration of macrolides to asymptomatic animals in the presence of diseased animals is used to improve the welfare of cattle and to decrease financial losses as a result of morbidities and mortalities (4, 10). However, antimicrobial use selects for antimicrobial-resistant (AMR) bacteria, including pathogens as well as harmless bacteria that can potentially act as a genetic reservoir of AMR gene determinants (4, 11). Excluding *M. bovis*, the genomes of BRD pathogens often contain integrative conjugative elements (ICE), mobile genetic elements that can harbor multiple AMR genes and encode the conjugation machinery required for transfer of ICE between BRD pathogens and to other bacteria (4, 9). The resulting multi-drug resistance (MDR) among some BRD pathogens containing ICE presents a significant challenge for the efficacy of antimicrobial therapy as a treatment for BRDClawson et al. (12) found that the gene *tet(H)*, which confers tetracycline resistance was present in all AMR *M. haemolytica* strains isolated from confirmed BRD cases, and was also frequently found in *P. multocida* (13) and *H. somni* ICE (14). Furthermore, *tet(H)* was adjacent to the transposase gene *tnpA*, a core ICE gene associated with increased minimum inhibitory antimicrobial concentrations in *M. haemolytica, H. somni*, and *P. multocida* (15).

Isolation of BRD pathogens by traditional culture methods and PCR verification of bacterial isolates (TC-PCR) has long been used to confirm disease outbreaks, but with several limitations (16). Traditional culture methods are time-consuming, requiring several days to obtain bacterial isolates, and some species such as *M. bovis* and *H. somni* grow poorly, a characteristic that may result in an under representation of the role of these pathogens in BRD (16–18). Therefore, new technologies continue to be evaluated to improve the diagnosis, early detection, and prognosis of BRD (2). In this study, recombinase polymerase amplification (RPA) is proposed as an alternative diagnostic application for BRD because of its simplicity, flexibility, multiplexing capabilities and rapidity (19). Originally developed by Piepenburg (20), RPA is a sensitive, isothermal DNA-based technology which utilizes primers and recombination proteins to generate DNA amplicons, that can either be visualized by gel electrophoresis or evaluated in real-time using fluorescent probes.

The aim of this study was to utilize RPA for detection of the four main bacterial pathogens associated with BRD, as well as AMR genes and ICE, and to develop multiple real-time RPA assays containing a competitive internal amplification control (IAC) to identify false negatives (21–23). Real-time RPA assays were tested on bovine deep nasopharyngeal swabs (DNPS) collected from cattle at feedlot arrival, to determine accuracy and sensitivity of RPA in comparison to TC-PCR for detection of BRD pathogens, and to its suitability for field-based detection.

## Methods

### DNA Extraction of Bacterial Strains

The strains used in this study are listed in Table 1. *M. haemolytica* and *P. multocida* strains were streaked onto tryptic soy agar containing sheep blood (TSA blood agar; Dalynn Biologicals, Calgary, AB, Canada) and incubated for 24 h at 37°C. *H. somni* strains were streaked onto TSA blood and incubated for 48 h at 37°C with 5% CO_2_. *M. bovis* was cultured by inoculating 1.5 ml pleuropneumonia-like organism broth (PPLO; brain heart infusion broth at 17.5 g per l, yeast extract at 25 g per l, and heat inactivated fetal horse serum at 200 mL per l) with a loop of glycerol stock culture. This starter culture was incubated at 37°C with 5% CO_2_ for 72–96 h. The entire 1.5 ml starter culture was then added to 30 ml PPLO broth and incubated for an additional 48 h.

**Table 1:** A list of control strains used in this study.

**Species**	**Strain**	**RPA assay**
*Mannheimia haemolytica* A1	ATCC BAA-410	*M. haemolytica (nmaA)*
*Mannheimia haemolytica* A6	ATCC 29697	*M. haemolytica (nmaA)*
*Pasteurella multocida*	CCUG 17976	*P. multocida (kmt1)*
*Histophilus somni*	ATCC 700025	*H. somni* (HS_0116)
*Mycoplasma bovis*	ATCC 25523	*M. bovis (uvrC)*
*Mannheimia haemolytica*	MH44 (9)	AMR, ICE (*tetH/tnpA*)
*Pasteurella multocida*	PM22 (9)	AMR, ICE (*tetH/tnpA*)
*Histophilus somni*	HS33 (9)	AMR, ICE (*tetH/tnpA*)

DNA was extracted from cultured cells using the DNeasy Blood & Tissue Kit (Qiagen, Toronto, ON, Canada) using the animal tissues spin-column protocol. For *M. haemolytica, P. multocida*, and *H. somni*, lysis of the cells was completed in Qiagen tissue lysis (ATL) buffer with proteinase K at 56°C for 3 h, followed by storage at 4°C overnight. The following day the protocol was resumed according to kit instructions with an additional wash buffer 2 (AW2) wash step. For *M. bovis*, the lysis step was reduced to 2 min and the full protocol was completed without overnight incubation.

### Preparation of Standard DNA

Extracted DNA was quantified using PicoGreen on the NanoDrop 3300 Fluorospectrometer (ThermoFisher Scientific, Ottawa, ON, Canada). The DNA was normalized to 10 ng/μl, and then to a 50,000 genome copies/μl stock and stored at −80°C. Calculation of DNA copy numbers per μl was based on the following formula: amount (copies/μl) = [DNA concentration (g/μl)/(bacterial genome length in base pairs × 660)] × 6.02 × 10^23^. The following genome sizes were used: *M. haemolytica* 2.6 Mbp, *P. multocida* 2.3 Mbp, *H. somni* 2.3 Mbp, and *M. bovis* 1 Mbp.

### Primer & Probe Design

Primers and probes were designed using Geneious 8.1.9 (Biomatters Ltd., Newark, NJ, USA) and verified using the NCBI BLAST nucleotide collection (nt/rt) reference sequence database (Table 2). The primers for *M. haemolytica* (*nmaA*) were designed for specificity to serotypes A1 and A6 because of their role as causative agents of BRD, while excluding serotype A2, a commensal of the bovine upper respiratory tract (12). Reference sequences used for primer design of each species-specific RPA include: *M. haemolytica* M42548 *nmaA* (GenBank: NC_021082.1), *H. somni* 2336 HS_0116 (GenBank: CP000947.1), *P. multocida Kmt1* (GenBank: FJ986389.1), and *M. bovis uvrC* (GenBank: AF003959.1).

**Table 2:** Primers and probes used in this study.

	**Target**	**Gene**	**Forward primer sequence**	**Reverse primer sequence**	**Amplicon size**	**Exo probe sequence (F = fluorophore; H = tetrahydrofuran; Q = quencher)**	**RPA assay type^**a**^**	**RPAKit^**b**^**
BRD targets	*Histophilus somni*	Hs_0116	CGTTTAATCCCATTGCGATCATTCCCCATT	ATACTATTGCATTCGGCGATTTTTCCGCTT	342	TATTCAAGTAGATGCAGATGGGCAGCATAAFHQAATTGATGTCAAGAA	1	B/E
	*Mannheimia haemolytica* A1 and A6	*nmaA*	TCAAAATGGCTCCCTTAGTTGAGGGCTTTA	AGTGGTTGCTGTATCGCCATGAACAAAAAT	254	TTCTGCTATTTTAGAAAAAATTCAACCTGTFHQTGCCGAATACAAAC	2	B/E
	*Mycoplasma bovis*	*uvrC*	ATGGTCCTTTTCCTTCTGGTTATGGAGCTA	TGGCTGCTTGATGCATTTTGTTAGTTAGTT	201	CAAAGACTATAACTTTTGGATTAATCAGTTFHAQAAAATTAAAGAAATT	2	B/E
	*Pasteurella multocida*	*kmt1*	GAACCGATTGCCGCGAAATTGAGTTTTATG	CCAACAAAACTGTGCTTTTCTTTGCCACAA	132		S	B
	*Pasteurella multocida*	*kmt1*	GAACCGATTGCCGCGAAATTGAGTTTTATG	CGAACTCGCCACTTTTTGTTTCATTTGGAC	417	ATTATTTTATGGCTCGTTGTGAGTGGGCTTGFHGGQAGTCTTTTATTT	1	E
	ICE	*tetH/tnpA*	CATCCACTAACTACGGCGCTGACATATCAA	TTGGTCCCCTTTTATTTGCCTTTATTTATA	318	TTAAGGGGTTGAAATAACAGCTTTAGGTGFHGQTTTTCTTTGGTGAA	S	B/E
	IAC	NA	Refer to Figure 2	Refer to Figure 2	Varies	GGGACGTGTATTTAACGTACTCGGAGAAAAFHQTGATTTGAATGAACCG	1, 2	E
AMR targets	Tilmicosin/tula-thromycin	*mph(E)*	TGGTATAAGTGAGCAATTGGAAACCCGCTA	TTGACCAATCAATAACGCCTGAAACAGCTC	155		S	B
	Tilmicosin/tula-thromycin	*msr(E)*	AGTCGCTATAACTGGATCGAATGGAACAGG	TTGAATATCATTCGCTCCGATCCCCATTGA	238		S	B
	Trimethoprim-Sulfadoxine	*sul2*	GGCCTATCTCAATGATATTCGCGGTTTTCC	GAATGCATAACGACGAGTTTGGCAGATGAT	90		S	B
	Florfenicol	*floR*	CTGGCGATGGATATTTATCTCCCTGTCGTT	ATCACCATATAGAGGCTCAACGTGAGTTGG	101		S	B
	Oxytetracycline	*tet(H)*	CAAAATCTGTCGATGATAATGCGCAAGGGA	ATAGCATAAAGTATTGCCCCCATCAGCCAT	166		S	B
	Tetracycline	*tetR*	CATTAAGCTCTATTGCGCATTTTACATTAG	CTTTAATACTGTTTCAAGTCCAGAGATCAT	215		S	B
	Tilmicosin/tula-thromycin	*Erm42*	GCCATGAATTTAAAAGTTCAAATGTGTCTA	TTGCTAAAGCTATGCAATATGTTAGTTTTG	283		S	B

a RPA assay type: 1 = multiplex, H. somni and P. multocida; 2 = multiplex, M. haemolytica and M. bovis; S = single-plex.

b * RPA kit: B = TwistAmp™ Basic Kit (conventional); E = TwistAmp™ Exo Kit (Real-time)*.

**Figure 1:**
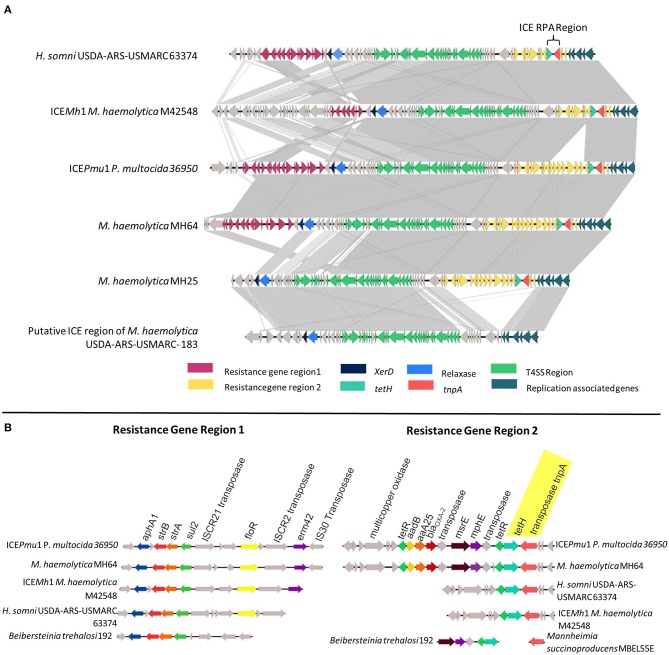
**(A)** Genomic comparison of the integrative conjugative element (ICE) regions of *H. somni* USDA-ARS-USMARC 63374, ICE*Mh*1, ICE*Pmu*1, *M. haemolytica* MH64, *M. haemolytica* MH25, and the putative ICE region of *M. haemolytica* USDA-ARS-USMARC-183. Genes are represented as arrows, with the arrowhead indicating the direction of transcription. Areas between ICEs shaded in light gray indicate regions of ≥67% sequence identity, while areas shaded in dark gray indicate ≥99% sequence identity. **(B)** Comparison of resistance gene regions 1 and 2 in representative bovine respiratory disease species with alignments to cassettes found in other bacterial species.

The genomes of five MDR *M. haemolytica* (MH25, MH30, MH64, MH69, MH76) and one *H. somni* (HS31) from our collection, as well as the published sequences of *P. multocida* 36950 ICE*Pmu*1 (GenBank: CP003022.1), *M. haemolytica* M42548 ICE*Mh*1 (GenBank: NC_021082.1), and *H. somni* USDA-ARS-USMARC 63374 (GenBank: CP018808.1) were utilized during the design of the ICE RPA assay (Figure 1). While ICEs differ among strains, the presence of *tet(H)* (conferring tetracycline resistance) was found in 100% of AMR *M. haemolytica* strains associated with BRD (12). While the *tet(H)* gene itself is prevalent among genomes of numerous bacterial species, within ICE, *tet(H*) is located adjacent to a transposase (*tnpA*) with a conserved sequence among ICE-containing strains of *M. haemolytica, P. multocida*, and *H. somni*. Therefore, the ICE RPA was designed to span a region of both *tet(H)* and *tnpA*, allowing for specific detection of AMR ICE-containing strains of all three important BRD pathogens (Figure 1).

### Species-Specific RPA Assays for BRD Pathogens & ICE

RPA reactions were performed in a total volume of 50 μl using the TwistAmp™ Basic Kit (TwistDX, Cambridge, UK). The reaction mixture included 420 nM each primer, 14 mM magnesium acetate, 29.5 μl rehydration buffer, 11.2 μl nuclease-free water, and 2 μl of bacterial DNA. A master mix was prepared containing all reagents except the DNA template and magnesium acetate, and then dispensed into 0.2 ml reaction tubes containing a dry enzyme pellet. Two microliters of DNA was added to each tube, followed by magnesium acetate into the tube lids and the lids were carefully closed. Reaction tubes were then vortexed and briefly centrifuged. Immediately thereafter, the reaction tubes were placed in an Eppendorf PCR thermocycler at 37°C to initiate the reaction. After 2 min the tubes were removed, briefly vortexed, centrifuged and then placed back into the thermocycler for another 28 min. Amplified RPA reactions were purified using the QIAquick PCR Purification Kit (Qiagen, Toronto, ON, Canada) automated on the QIAcube (Qiagen, Toronto, ON, Canada). Following purification, RPA products were electrophoresed on 2% (w/v) agarose gels containing ethidium bromide, and visualized using a fluorescence imager (FluorChem FC2; Alpha Innotech, San Leandro, CA, USA).

Each species-specific RPA assay, including multiplex and real-time assays were screened for inclusivity against 36 representative isolates of each of the four target species (*n* = 144). The *M. haemolytica* isolates represented both serotypes A1 and A6 and encompassed 35 different pulsed field gel electrophoresis (PFGE) profiles. Isolates were obtained from lung tissues of BRD mortalities, collected in both Canada and the USA (9, 15, 24). Arising from the same studies, *P. multocida* and *H. somni* isolates belonged to 31 and 21 PFGE types, respectively. The *M. bovis* isolates were collected from the Stanford et al. (15) study and consisted of 27 different PFGE profiles.

A total of 66 bacterial strains (Table 3) belonging to BRD pathogens, closely related species, or other species known to be present in the upper and lower bovine respiratory tract were used to test the specificity of the BRD target RPA assays using the basic kit (Table 2). Bacterial strains were purchased from the American Type Culture Collection (ATCC), Culture Collection University of Gothenburg (CCUG), or obtained from a collaborating laboratory (25). DNA was extracted using the DNeasy Blood & Tissue Kit (Qiagen, Toronto, ON, Canada) with appropriate protocols for Gram positive and Gram negative bacteria.

**Table 3:** A list of strains used for recombinase polymerase amplification specificity testing.

**Target strains**	**Species**	**Strain/origin**
	*Mannheimia haemolytica A1*	ATCC BAA-410
	*Mannheimia haemolytica A6*	ATCC 29697
	*Pasteurella multocida*	CCUG 17976B
	*Histophilus somni*	ATCC 700025
	*Mycoplasma bovis*	ATCC 25523
Non-target strains	*Mannheimia haemolytica A7*	ATCC 29698
	*Mannheimia haemolytica A9*	ATCC 29700
	*Mannheimia haemolytica A2*	ATCC 33396
	*Mannheimia varigena* (2 strains)	CCUG 38475, CCUG 38462
	*Mannheimia*	CCUG 38461
	*Mannheimia granulomatis*	CCUG 45422
	*Mannheimia ruminalis* (2 strains)	CCUG 38470, CCUG 38466
	*Mannheimia glucosida (7 strains)*	CCUG 28376, CCUG 38458, CCUG 38467, CCUG 38460, CCUG 28375, CCUG 38459, CCUG 38456
	*Pasteurella canis*	ATCC 43326
	*Haemophilus influenza* (2 strains)	ATCC 33391, ATCC 10211
	*Haemophilus parasuis*	ATCC 19417
	*Mycoplasma bovirhinis*	ATCC 27748
	*Mycoplasma alkalescens*	ATCC 29103
	*Mycoplasma canadense*	ATCC 29418
	*Mycoplasma bovigenitalium*	ATCC 19852
	*Mycoplasma bovoculi*	ATCC 29104
	*Mycoplasma californicum*	ATCC 33461
	*Mycoplasma conjunctivae*	ATCC 25834
	*Mycoplasma arginini*	ATCC 23243
	*Mycoplasma canis*	ATCC 19525
	*Mycoplasma ovipneumoniae*	ATCC 29419
	*Trueperella pyogenes*	ATCC 19411
	*Moraxella bovoculi/lacunata*	(25)
	*Moraxella bovoculi/bovis*	(25)
	*Moraxella osloensis*	(25)
	*Psychrobacter pulmonis/faecalis*	(25)
	*Psychrobacter sanguinis*	(25)
	*Pseudomonas aeruginosa* (2 strains)	ATCC 27853, ATCC 10145
	*Acinetobacter baumannii*	ATCC 17978
	*Acinetobacter lwoffii*	(25)
	*Acinetobacter bouvetti*	(25)
	*Acinetobacter calcoaceticus/ oleivorans /juni*	(25)
	*Escherichia coli* (2 strains)	ATCC 35218, ATCC 25922
	*Streptococcus pneumoniae*	ATCC 33400
	*Streptococcus bovis*	ATCC 33317
	*Staphylococcus aureus* (3 strains)	ATCC 35556, ATCC 29213, ATCC 29740
	*Clostridium butyricum*	ATCC 19398
	*Clostridium difficile*	ATCC 9689
	*Actinobacillus succinogenes*	ATCC 55618
	*Bacillus atrophaeus*	ATCC 9372
	*Bacillus cereus*	ATCC 10702
	*Bacillus licheniformis*	ATCC 14580
	*Bacillus mycoides*	ATCC 6462
	*Bacillus subtilis*	ATCC 6633
	*Bacillus thuringiensis*	ATCC 33679
	*Leucobacter chromiireducens*	ATCC BAA-1336
	*Bibersteinia trehalosi* (2 strains)	CCUG 27190, CCUG 37711

Similarly for the ICE RPA, reactions were prepared as described above. Specificity of the ICE target was evaluated using the three ICE control strains from our collection (Table 1) as well as an additional 22 sequenced strains (belonging to *M. haemolytica, P. multocida* and *H. somni*), 11 with and 11 without ICE.

### TwistAmp™ Basic Kit Multiplex RPA Assay

A multiplex RPA using the TwistAmp™ Basic Kit (TwistDX, Cambridge, UK) was developed for the simultaneous amplification of all four of the BRD pathogens. Reactions were prepared as described (section Species-Specific RPA Assays for BRD Pathogens & ICE) with each of the 8 primers included at 120 nM.

### AMR Gene RPA Assays Using TwistAmp™ Basic Kit

Seven RPA assays were designed for AMR genes (*tetH, tetR, msrE, mphE, sul2, floR, erm42*). Primers are listed in Table 2 and reactions were prepared using the TwistAmp™ Basic Kit (TwistDX, Cambridge, UK) as described in section Species-Specific RPA Assays for BRD Pathogens & ICE. AMR gene RPA assays were verified using the sequenced strains listed in Table 1 (9).

### Design of IAC for Multiplex Real-Time RPA

**Figure 2:**
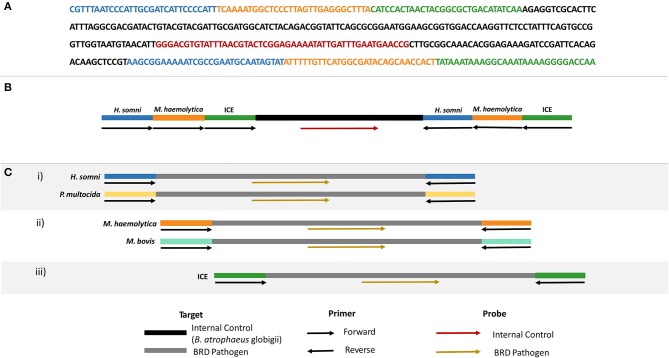
**(A)** Nucleotide sequence of the internal amplification control (IAC), including primer binding sites for *H. somni* (blue), *M. haemolytica* (orange), and ICE (green), and the internal control probe binding site (red). Backbone sequence belonging to *B. atrophaeus* subsp. globigii indicated in black. **(B)** Schematic representation of the oligonucleotide primer and probe locations on the IAC. **(C)** Schematic representation of each real-time RPA assay to be used with the IAC.

A competitive internal amplification control (IAC) was designed for use in multiplex real-time RPA and ICE RPA assays so that target primers also amplified the IAC, eliminating the need for additional primers specific for an internal control (Figure 2). Note that only one set of the target primers amplified the IAC, and therefore a positive control is still required as a verification for the other target primer set. The IAC template consisted of a sequenced region unique to *Bacillus atrophaeus* subsp. *globigii* (26, 27) containing a binding site for the IAC probe, and flanked by the primer sequences for *H. somni, M. haemolytica*, and ICE. The IAC was synthesized and inserted into a plasmid vector (pCR2.1) by Eurofins Genomics (Toronto, ON, Canada). The IAC plasmids were transformed into *E. coli* DH5α cells (Invitrogen, Thermo Fisher Scientific, Waltham, MA, USA). Following plasmid purification using the QIAprep Spin Miniprep Kit (Qiagen, Toronto, ON, Canada), plasmid DNA was quantified by PicoGreen (Invitrogen, Thermo Fisher Scientific, Waltham, MA, USA), normalized to 1 × 10^8^ copies/μl and serially diluted to 5 × 10^2^ copies/μl for use in real-time RPA assays.

### Real-Time RPA Assays

Three real-time RPA assays were developed: (i) *P. multocida* and *H. somni* multiplex, (ii) *M. haemolytica* and *M. bovis* multiplex, and (iii) ICE RPA assay (Figure 2C). Real-time RPA was completed using the TwistAmp™ Exo Kit (TwistDX, Cambridge, UK). Reactions for ICE contained 420 nM of each ICE primer, 78 nM ICE probe, 24 nM internal control probe, 14 mM magnesium acetate, 29.5 μl rehydration buffer, 11.3 μl nuclease-free water, 1 × 10^3^ genome copies per reaction internal control plasmid, and 2 μl of bacterial or sample DNA. Multiplex RPA reactions for *M. haemolytica* and *M. bovis* were prepared in the same way with the following modifications: 210 nM each primer, 45 nM each of *M. haemolytica* and *M. bovis* probe, and 30 nM internal control probe. Finally, for the *P. multocida* and *H. somni* multiplex RPA, reactions contained 190 nM *P. multocida* primers, 230 nM *H. somni* primers, 42.75 nM *P. multocida* probe, 52.25 nM *H. somni* probe, and 25 nM internal control probe, with all other reaction components being the same as for the ICE real-time assay. Reactions were prepared as described in section Species-Specific RPA Assays for BRD Pathogens & ICE with the following modifications: a magnetic bead was dispensed into each reaction tube immediately following the addition of master mix, and reaction tubes were placed in a T16-ISO instrument (TwistDX, Cambridge, UK) at 37°C for 33 min. Positive amplification was asserted when the fluorescence measured over 200 mV for 60 s.

The limit of detection (LOD) was determined for each real-time RPA using dilutions of genomic DNA (ranging from 1 to 1000 genome copies/reaction). Five reactions were prepared per DNA template concentration, with each run repeated 4 times, for a total of 20 reactions per dilution.

### Using RPA on Bovine Nasal Swabs

The ICE-specific real-time RPA assay, *M. haemolytica*/*M. bovis*, and *P. multocida/H. somni* multiplex real-time assays were tested using 100 DNPS collected from feedlot cattle, which were also screened for BRD pathogens using TC-PCR. Samples were obtained under the supervision of a trained veterinarian and the protocol was reviewed and approved by the Lethbridge Research Center Animal Care Committees in accordance with guidelines of the Canadian Council on Animal Care (28). Consent for sampling of the cattle was also obtained from the owners.

Swabs for RPA testing were selected based on PCR-verified culture data, including those positive for any combination of the four bacterial pathogens as well as samples which were culture negative for all four pathogens. Briefly, DNPS were placed into 1 ml brain heart infusion broth containing 20% glycerol (Dalynn Biologicals, Calgary, AB) and vortexed for 1 min. Methods for TC-PCR detection of *M. haemolytica, P. multocida*, and *H. somni* were identical to those described by Stanford et al. (15) with the following modifications: 100 μl of DNPS suspension was plated for *M. haemolytica* and *P. multocida*, 50 μl each of undiluted DNPS suspension and 10^−1^ dilution were plated for *H. somni* and incubated for 48 h. Methods for TC-PCR detection of *M. bovis* were completed as described by Andrés-Lasheras et al. (29). DNA was obtained from a 300 μl aliquot of DNPS suspension using the DNeasy Blood and Tissue kit (Qiagen, Toronto, ON, Canada). RPA reaction mixtures contained primers and probes at concentrations described in section Real-time RPA Assays, with 10 μl DNA sample, and 1.3 μl nuclease-free water.

### Statistical Analysis

The LOD values for each RPA at a probability of detection of 95% were estimated by Probit regression analysis using Microsoft Excel (2016). Results of real-time, multiplex RPA and TC-PCR were compared by measuring the degree of agreement and kappa coefficient (*k*) (Table 4).

**Table 4:** Comparison of traditional culture - PCR (T-PCR) and recombinanse polymerase amplificaton (RPA) for detection of bovine respiratory disease pathogens in deep nasopharyngeal swab samples.

	***M. haemolytica***			***M. bovis***			***P. multocida***			***H. somni***			**Overall**		
	**TC-PCR+**	**TC-PCR−**	**Total**	**TC-PCR+**	**TC-PCR−**	**Total**	**TC-PCR+**	**TC-PCR−**	**Total**	**TC-PCR+**	**TC-PCR−**	**Total**	**TC-PCR+**	**TC-PCR−**	**Total**
RPA +	32	2	34	44	14	58	28	7	35	27	13	40	131	36	167
RPA −	11	55	66	2	40	42	19	46	65	8	52	60	40	193	233
Total	43	57	100	46	54	100	47	53	100	35	65	100	171	229	400
	Agr: 87%	*k*: 0.728	89%^*^	Agr: 84%	*k*: 0.684	98%^*^	Agr: 74%	*k*: 0.470	81%^*^	Agr: 79%	*k*: 0.553	92%^*^	Agr: 81%	*k*: 0.611	90%^*^

+, positive; −, negative; Agr, agreement; k, kappa coefficient.

Agreement, [RPA positive, TC-PCR positive + RPA negative, TC-PCR negative]/total number of instances.

* *Total % of instances of pathogen presence where RPA matched or exceeded detection by TC-PCR*.

## Results

**Figure 3:**
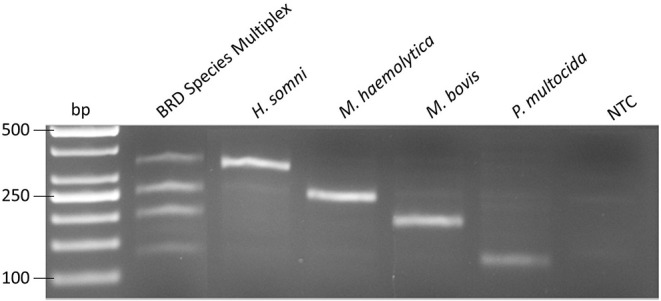
Amplification by multiplex recombinase polymerase amplification for all 4 bovine respiratory disease pathogens, *H. somni, M. haemolytica, M. bovis*, and *P. multocida* at 5 × 10^2^ genome copies. NTC, no template control.

Using the TwistAmp™ Basic kit, RPA assays were optimized for ICE and each BRD species individually (*M. haemolytica, P. multocida, H. somni*, and *M. bovis*), as well as being used in a conventional multiplex containing all four BRD targets (Figure 3). RPA assays demonstrated 100% inclusivity and analytical specificity, as all 36 strains of each species were successfully identified in each species-specific RPA assay, and the 5 target strains were successfully detected (Table 3), while none of the 61 non-target strains were detected. Additionally, seven single-plex RPA assays were developed for AMR genes (*tetH, tetR, msrE, mphE, sul2, floR, erm42*). Positive and negative amplification was verified for each AMR gene assay using sequenced AMR strains (data not shown).

**Figure 4:**
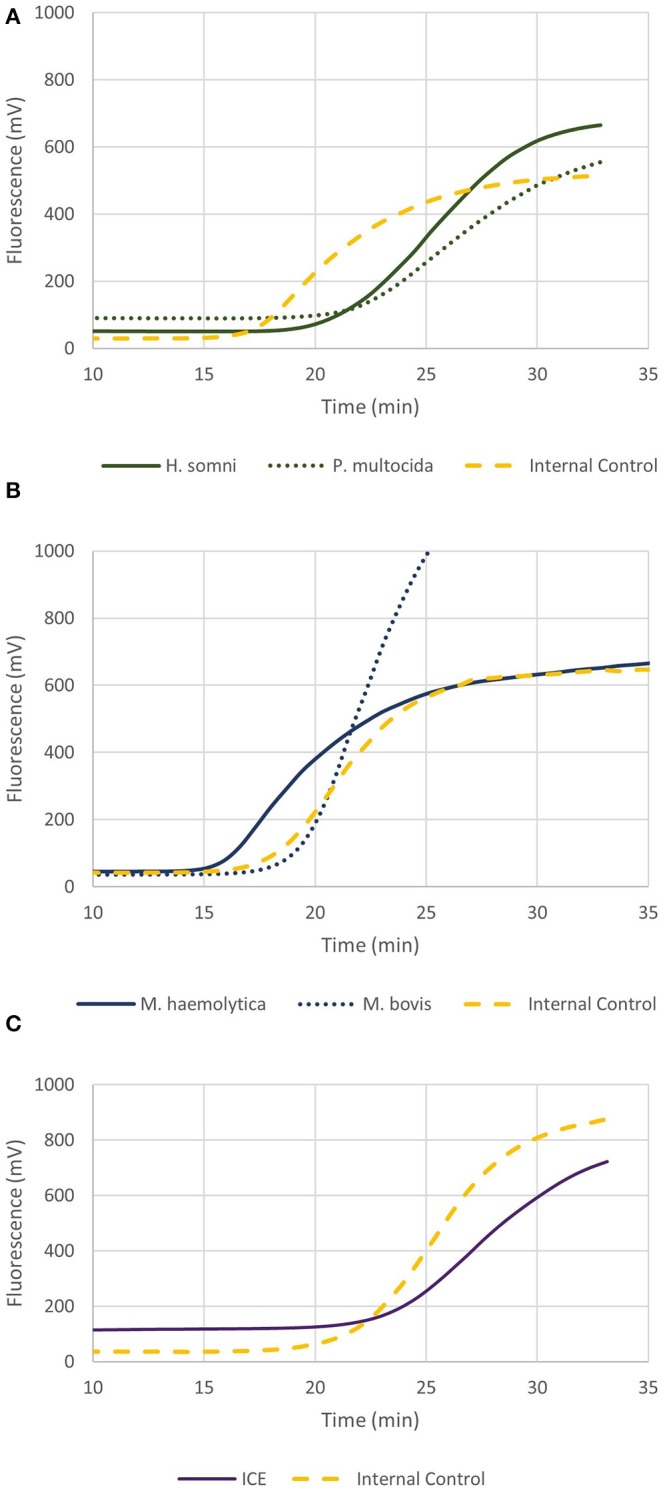
Graphs depicting recombinase polymerase amplification (RPA) over time of 2 × 10^2^ genome copies for **(A)** *P. multocida* and *H. somni* multiplex, **(B)** *M. haemolytica* and *M. bovis* multiplex, and **(C)** ICE RPA. Each assay included the internal amplification control (1 × 10^3^ genome copies) designed in this study.

The real-time multiplex RPA assays are shown in Figures 4A,B, for *P. multocida*/*H. somni* and *M. haemolytica*/*M. bovis*, respectively. Each assay contained the IAC and the LOD was 161 and 40 genome copies, respectively, for *P. multocida/H. somni* and *M. haemolytica*/*M. bovis* assays. As few as 103 and 7 genome copies, could be detected in 50% of cases for *P. multocida/H. somni* and *M. haemolytica*/*M. bovis*, respectively.

**Figure 5:**
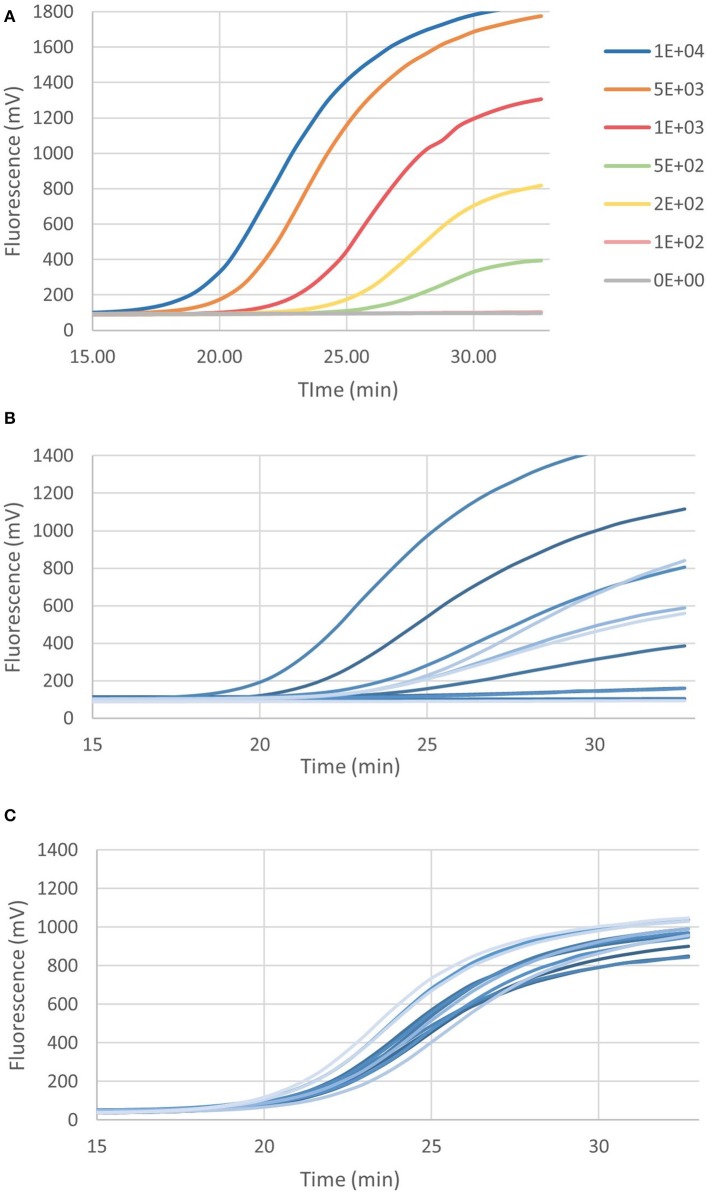
**(A)** The recombinase polymerase amplification over time of ICE at decreasing genome copies (*M. haemolytica* MH44). **(B)** Example of ICE amplification from bovine nasal swabs, and **(C)** amplification of internal amplification control (1 × 10^3^ genome copies) in bovine nasal swabs.

Figure 4C shows the real-time RPA assay for a region of the ICE specific to *M. haemolytica, P. multocida*, and *H. somni*, along with the IAC. The LOD for the ICE RPA was 134 genome copies per reaction (95% confidence interval). In 50% of cases, as few as 97 genome copies per reaction could be detected. Figure 5A illustrates the real-time RPA amplification of ICE using decreasing concentrations of genomic DNA template (1 × 10^4^ to 1 × 10^2^ copies/reaction).

Bovine DNPS samples (*n* = 100) were screened for ICEs and BRD pathogens using the ICE RPA and real-time multiplex RPA assays for BRD pathogens. RPA results were compared to data collected by TC-PCR for each BRD species. Figure 5B shows an example of the amplification results of the ICE RPA using DNPS samples collected from individual cattle upon arrival at the feedlot. The IAC successfully amplified in DNPS reactions (Figure 5C). Based on TC-PCR data, among the 100 bovine DNPS swabs selected for this study, each contained 0 to 4 of the selected members of the bacterial BRD complex, denoting a total of 131 instances of BRD pathogens. RPA exhibited 81% agreement (kappa coefficient*, k* = 0.611) with the TC-PCR data, while in an additional 36 instances, pathogens were detected by RPA, and in 40 instances detected by TC-PCR only (Table 4). The results showed that RPA had a positive rate that was similar to that of TC-PCR (Table 4), with detection of *M. bovis* and *H. somni* being higher by RPA, and *M. haemolytica* and *P. multocida* lower by RPA than as result of culture from DNPS. Positive rates were as follows, for TC-PCR vs. RPA, respectively: 43 vs. 34% for *M. haemolytica*, 46 vs. 58% for *M. bovis*, 47 vs. 35% for *P. multocida*, and 35 vs. 40% for *H. somni*. Agreement of RPA with culture data for *P. multocida* was 74% (*k* = 0.470), *H. somni* was 79% (*k* = 0.553), *M. bovis* was 84% (*k* = 0.684), and *M. haemolytica* was 87% (*k* = 0.728). Results in which RPA either agreed with or exceeded pathogen detection over culture methods accounted for 81, 89, 92, and 98% of cases for *P. multocida, M. haemolytica, H. somni*, and *M. bovis*, respectively. ICE was detected in 55% (*n* = 55) of the bovine nasal swabs tested. Of the swabs positive for ICE, 91% (*n* = 50) were also positive for one or more of the BRD-associated pathogens by RPA and/or TC-PCR.

## Discussion

In this study, RPA assays were developed to detect four bacterial BRD pathogens (*M. haemolytica, M. bovis, H. somni*, and *P. multocida*), seven AMR genes, and a region of ICE associated with BRD pathogens. Furthermore, detection of *M. haemolytica* was specific to serotypes A1 and A6, those most commonly associated with disease, while excluding all other serotypes, including A2 a common bovine commensal (30, 31). Beker et al. (13) developed a multiplex PCR assay targeting four conserved core genes required for integration and maintenance of ICE structures within the *Pasteurellaceae* family and demonstrated relevance of this assay to detecting these elements in *P. multocida* and *M. haemolytica* (13). Furthermore, RPA has recently been utilized for detection of *P. multocida* in cattle (32). However, to our knowledge, this is the first study to develop and apply RPA for detecting four major bacterial BRD pathogen species in multiplex and real-time formats, and BRD pathogen-associated with ICEs in bovine DNPS.

A conventional multiplex RPA assay was designed using the TwistAmp™ basic kit for simultaneous amplification of the four major BRD bacterial species. While this assay is useful for verification of presumptive positive isolates identified from culture methods in a laboratory setting, all RPA assays using the TwistAmp™ basic kit require post-amplification clean up to remove excess proteins, and gel electrophoresis for visualization of amplified products, a procedure not easily achieved outside of a laboratory (33). In an effort to develop RPA assays for use in the field, RPA assays were modified for real-time detection using the TwistAmp™ Exo kit and T16-ISO instrument (TwistDX, Cambridge, UK). In comparison to real-time PCR, the RPA instrument cannot run as many reactions at a time, nor are the results quantitative. However, results are achieved within 20–30 min vs. 1.5–2 h with real-time PCR. The procedure exhibits similar sensitivity, and the instrument is substantially smaller and less expensive than a real-time PCR machine making it more suitable for a field application (33).

A real-time RPA assay for ICEs and two multiplex real-time RPAs were developed, each containing a competitive IAC. The addition of an IAC has been shown to avoid false-negatives (22, 23, 34). As opposed to a non-competitive IAC, a competitive IAC is co-amplified simultaneously with the target by the same primer set (23). By using a competitive IAC, the target and IAC are amplified by the same primers under the same conditions, reducing the need for an additional primer set, maximizing the quantity of the target primer. A competitive IAC also reduces the risk of undesirable interactions among the target primers and an additional control primer set (23). A limitation of this approach is the requirement for exogenous synthetic DNA.

The LOD was 161 and 40 genome copies per reaction for *P. multocida/H. somni* and *M. haemolytica*/*M. bovis* assays, respectively, and 134 genome copies for ICE. Limits of detection were similar to other published RPA and multiplex RPA assays (27, 32, 35, 36). Sensitivity of RPA depends greatly on primer and probe design, but design software and recommendations are currently lacking (19). As a result, several RPA primer and probe sets must be screened in order to determine the optimal combination (19). Multiplexing offers additional challenges, as competition among primer sets for recombinase proteins can result one target preventing the amplification of another (37).

The real-time RPA assay for ICEs amplified a region conserved among three of the four BRD pathogens targeted (*M. haemolytica, H. somni*, and *P. multocida*). An ICE is a mobile genetic element, transferred via conjugation between bacteria of the same or different species (9). ICEs may differ among species as well as within strains of the same species, containing as few as 1 to as many as 12 or more AMR genes (12). The gene *tet*(H), responsible for resistance to tetracycline has been associated with plasmids and chromosomal DNA, and also on a transposon-like element of *P. multocida* known as Tn5706 (38). The presence of *tet*(H) in ICEs is frequent among AMR *M. haemolytica, H. somni*, and *P. multocida* strains (12, 14, 39, 40). Within the ICE, *tet(H*) is located directly next to a transposase (*tnpA*) with a conserved sequence among ICE-containing strains of *M. haemolytica, P. multocida* and *H. somni*. Furthermore, *tet*(H) has only been reported in members of the *Pasteurellaceae* (39). Therefore, the ICE RPA was designed to span a region of both *tet(H)* and *tnpA* allowing for specific detection of three of the bacterial BRD bacterial pathogen that can potentially harbor AMR-ICE.

The bovine DNPS used in this study were collected from cattle upon arrival at the feedlot. Arrival at the feedlot is a particularly stressful period for cattle, which often involves transportation over long distances, and comingling of cattle, increasing transmission of BRD agents among members of the herd (6). While traditional culture methods are the standard for confirmation of BRD infection, they are not without limitations. Bovine nasal swabs inoculated onto agar plates can easily become overgrown by non-target bacteria, making it difficult to visually identify and isolate target species. Of the four bacterial BRD pathogens, *P. multocida* and *M. haemolytica* are most easily identified on the basis of morphology, however this approach is highly subjective. While *H. somni* also has a distinct morphology, it is difficult to culture and is easily overgrown as it requires twice the incubation period of *P. multocida* and *M. haemolytica* (16). *M. bovis* is even more challenging to culture as it requires a significantly longer to grow than other BRD pathogens, and must be cultured under humidified, microaerophilic conditions (16).

Detection of BRD species using multiplex real-time RPA showed a strong correlation with TC-PCR (90%). A greater number of swabs containing *M. bovis* and *H. somni* were detected by RPA than by TC-PCR, likely due to the aforementioned challenges associated with culturing these species in the laboratory. In contrast, fewer swabs were identified containing *M. haemolytica* and *P. multocida* by RPA than by TC-PCR. Likely, this is due to the ease with which these two species are cultured, and their distinct morphologies on laboratory media, aiding identification even when cell numbers are low. Culture-positive results for serotype A2 during TC-PCR were excluded as a positive result for *M. haemolytica* during data interpretation, and therefore is not a reason for the lower detection by RPA. However, RPA identified the presence of ~10% more bacterial pathogens (36 instances) in swabs than TC-PCR, reflecting the greater sensitivity of RPA over traditional culture methods.

The ICE RPA assay was utilized to screen DNPS, because unlike the AMR gene RPA assays, this particular target is specific to all three BRD bacterial species, while also serving as an indicator of AMR and potential MDR. ICE was detected among 55% (*n* = 55) of the nasal swabs tested in this study. No BRD pathogens were detected in 9% of ICE-positive DNPS samples. Due to the transmissible nature of ICE, this suggests that BRD pathogens may be transferring ICE to other bacterial species (13, 41). A closely related species, *Bibersteinia trehalosi*, as well as *Moraxella* and *Acinetobacter* may also contain ICE (9, 31).

In this study, RPA was demonstrated to be a useful technology for detection of BRD pathogens and ICE from bovine nasal swabs. Advantages of RPA over polymerase chain reaction (PCR) and other isothermal technologies include simplified instrumentation amenable for field-based studies and reduced costs (19). Furthermore, detection by RPA is sensitive, and results can be obtained in real-time in <30 min (19). Similar to other molecular based techniques, detecting the AMR profile of BRD agents by RPA does not eliminate the need for culture methods. However, conventionally, it takes 2–5 days to confirm identity of BRD agents in a laboratory setting whereas RPA can accomplish this same feat in 1–2 h. Furthermore, RPA is more tolerant to inhibitors and background DNA than PCR (33). The robustness of RPA in the presence of traditional inhibitors facilitates amplification from crude extracts, which is not achievable using PCR (37).

Diagnosis of BRD in live cattle remains difficult, since there is no gold standard to define a BRD infection (2). Because many of the BRD pathogens are also commensals, their presence alone is not necessarily an indicator of disease without other predisposing environmental factors, physiologic stressors, or concurrent (viral) infections (6, 42). This affects the ability to accurately evaluate methods or technologies for diagnosis of BRD (2). A greater understanding of the virulence mechanisms of the infecting bacteria and pathogenesis is needed (6).

Further research is required to optimize RPA technology for BRD detection in the feedlot. Specifically, a method for obtaining a high yield and quality of nucleic acids from bovine nasal swabs without the use of a commercial kit will be required. Further refinement of RPA assays to enhance sensitivity and multiplexing capability would also be beneficial. Finally, a deeper understanding of the gene mechanisms associated with virulence and antimicrobial resistance of BRD pathogens may lead to identification of additional signature genes to further improve the utility of RPA.

## Conclusion

RPA is a sensitive, specific and accurate method which detected 4 major BRD bacterial agents in deed nasal swabs collected from feedlot cattle. Furthermore, RPA was capable of detecting ICE from MDR *M. haemolytica, P. multocida*, and *H. somni* strains, which may contribute to dissemination of AMR and virulence genes among BRD pathogens. As compared to conventional approaches for detecting BRD pathogens, RPA is affordable, fast, and easily modified for real-time field-based detection. Further studies are required to evaluate performance of RPA in field settings. Additional study linking detected pathogens to clinical BRD as well as signature genes responsible for AMR profiles would enable RPA-guided selection of effective antimicrobial treatments by the beef industry, reducing antimicrobial usage by minimizing the need for repeated treatments due to AMR.

## Data Availability Statement

All datasets generated for this study are included in the article/supplementary material.

## Ethics Statement

The animal study was reviewed and approved by Lethbridge Research Centre Animal Care Committees in accordance with guidelines of the Canadian Council on Animal Care (2009). Written informed consent was obtained from the owners for the participation of their animals in this study.

## Author Contributions

YN, KS, TA, SC, BR, and TM conceived and designed the study. RD, MB, MGB, and KA provided expertise in RPA technology and assisted in design and development of RPA assays. CC performed laboratory activities. RZ and CC worked on analysis of the sequencing data. CC performed other statistical analyses and wrote the first draft of the manuscript. All authors revised the manuscript and gave approval for the final version to be published.

## Conflict of Interest

The authors declare that the research was conducted in the absence of any commercial or financial relationships that could be construed as a potential conflict of interest.
